# Prediction of early postoperative complications and transfusion risk after lumbar spinal stenosis surgery in geriatric patients: machine learning approach based on comprehensive geriatric assessment

**DOI:** 10.1186/s12911-025-03125-1

**Published:** 2025-07-28

**Authors:** Wounsuk Rhee, Sam Yeol Chang, Bong-Soon Chang, Hyoungmin Kim

**Affiliations:** 1 Ministry of Health and Welfare, Government of the Republic of Korea, 13, Doum 4-ro, Sejong, 30113 Republic of Korea; 2 Siebel School of Computing and Data Science, University of Illinois Urbana-Champaign, 201 N. Goodwin Avenue, Urbana, IL 61801 USA; 3 Healthcare AI Research Institute, Seoul National University Hospital, 101, Daehak-ro, Jongno-gu, Seoul, 03080 Republic of Korea; 4 Department of Orthopedic Surgery, Seoul National University College of Medicine, 103, Daehak-ro, Jongno-gu, Seoul, 03080 Republic of Korea; 5 Department of Orthopedic Surgery, Seoul National University Hospital, 101, Daehak-ro, Jongno-gu, Seoul, 03080 Republic of Korea

**Keywords:** Lumbar spinal stenosis, Comprehensive geriatric analysis, Postoperative complications, Transfusion, Personalized medicine, Artificial intelligence, Machine learning, Model explainability

## Abstract

**Background:**

Lumbar spinal stenosis is one of the most common surgery-requiring conditions of the spine in the aged population. As elderly patients often present with multiple comorbidities and limited physiological reserve, individualized risk assessment using comprehensive geriatric assessment is crucial for optimizing surgical outcomes.

**Methods:**

Patients 65 years or older who underwent elective surgery for lumbar spinal stenosis between June 2015 and December 2018 were prospectively enrolled, resulting in 261 eligible patients of age 72.3 ± 4.8 years (male 108, female 153). Twenty-seven experienced complications of Clavien-Dindo grade 2 or higher within 30 days, and 79 received transfusion during hospital stay. The cohort was split into train-validation (*n* = 208) and test (*n* = 53) sets. A total of 48 features, including demographics, comorbidity, nutrition, and perioperative status, were collected. Logistic regression, support vector machine (SVM), random forest, XGBoost, and LightGBM were trained using five-fold cross-validation. AUROC and AUPRC were considered the primary performance metrics, and the results were compared with those estimated with ACS-NSQIP scoring system. A set of *Compact* models incorporating a smaller number of features was also trained, and SHAP analysis was conducted to evaluate the models’ interpretability.

**Results:**

The reduced number of features did not result in the drop of AUROC and AUPRC for all machine learning algorithms (*P* > 0.05). when compared to the ACS-NSQIP scoring system, which achieved a test AUROC of 0.38 (95% confidence interval [CI], 0.13–0.73) and 0.22 (95% CI, 0.10–0.36) on the first two tasks, the *Compact* model showed significantly greater AUROC values nearing or surpassing 0.90. Decision tree-based algorithms demonstrated larger test AUROC than logistic regression and generally agreed on the most influential features for each task.

**Conclusions:**

Advanced machine learning models have consistently shown greater performance and interpretability over conventional methodologies, implying their potential for a more individualized risk assessment of the aged population.

**Trial registration:**

Not applicable as this research is not a clinical trial.

**Supplementary Information:**

The online version contains supplementary material available at 10.1186/s12911-025-03125-1.

## Introduction

Lumbar spinal stenosis (LSS) is one of the most common degenerative diseases of the spine, often leading to chronic pain and gait disturbances that significantly impair quality of life [1–3]. When symptoms progress, surgical decompression with or without spinal fusion becomes necessary to alleviate neurological deficits and restore function [1–4]. LSS is particularly prevalent in the elderly population, with its incidence increasing with age due to progressive degenerative changes in the spine [1, 5]. Elderly patients often present with multiple comorbidities and reduced physiological reserves, posing a significant risk of perioperative complications, including surgical site infections, cardiopulmonary events, and prolonged recovery times [5–10]. Therefore, accurate risk stratification in this population is crucial for optimizing surgical decision-making and improving postoperative outcomes.

Various scoring systems have been utilized to comprehensively assess the health status of elderly patients and predict postoperative complications and transfusion risk. Commonly used tools include the American Society of Anesthesiologists Physical Classification System (ASA classification), the American College of Surgeons National Surgical Quality Improvement Program (ACS-NSQIP) surgical risk calculator, and the Charlson Comorbidity Index (CCI), all of which provide structured risk assessments based on preoperative patient characteristics [11–13]. Particularly for patients undergoing surgical intervention of LSS, comprehensive geriatric assessment (CGA) has been proposed to evaluate the risk of early postoperative complications [5]. Unlike traditional methods, CGA aims to integrate multiple dimensions of health, including frailty, activities of daily living (ADL), mini-mental state examination (MMSE), nutritional status, and medication burden [9, 10, 14–16].

However, despite their clinical effectiveness, the aforementioned methods rely on a limited number of predefined variables and simple statistical models, which may fail to adequately account for the complexity of health conditions in elderly patients. Contrastingly, machine learning (ML) offers a data-driven approach that can integrate numerous clinical factors and capture intricate, nonlinear relationships among them [17, 18]. Among various techniques, decision tree-based algorithms, including random forests and gradient boosting machines have gained attention due to their robustness and interpretability [19–22].

Recently, a growing body of research has demonstrated the effectiveness and potential of ML in the context of geriatric surgery. For instance, in gastrointestinal cancer surgeries, ML models have outperformed traditional bedside scoring systems in predicting postoperative morbidity and mortality [23]. Similarly, several studies have reported the superior performance of ML algorithms in predicting delirium, a common postoperative complication among elderly patients [24, 25]. Furthermore, there have been efforts to apply ML to forecast cardiovascular events in non-cardiac surgeries, as well as to predict the onset of acute kidney injury [26, 27]. Collectively, these findings highlight the substantial potential of ML as a powerful tool for personalized risk assessment and decision-making in elderly patients, who often present with diverse and complex clinical profiles. In the context of spinal surgery, ML have been applied to various prediction tasks, including estimating prolonged length of hospital stay, predicting the likelihood of blood transfusion after adult spinal deformity surgery, and forecasting changes in patient-reported outcomes such as the SRS-22R questionnaire [28–31]. However, most of the preliminary studies were not focused on the geriatric population, and we aimed to specify the cohort as the elderly are more vulnerable to adverse events after surgery.

Using a prospectively collected cohort of elderly patients undergoing surgery for LSS, we develop and evaluate ML models predicting early postoperative complications and the need for blood transfusion during the hospitalization period. By leveraging ML techniques, we seek to improve the accuracy of perioperative risk assessment compared to traditional methods, particularly the ACS-NSQIP scoring system. Furthermore, Shapley additive explanations (SHAP) analysis is performed to identify key risk factors contributing to early postoperative outcomes and, if possible, provide clinically actionable insights [32].

## Materials and methods

### Patient cohort

Patients aged 65 years or older who underwent any type of elective surgery for LSS between June 2015 and December 2018 were prospectively enrolled. Those treated for spinal deformities or stenosis at non-lumbar levels, as well as patients requiring emergency operations, were excluded to ensure a more homogeneous study population. The study was approved by the Institutional Review Board (IRB) of our organization, and informed consent was obtained from all participants.

### Data acquisition

A wide range of patient characteristics were collected one day before surgery. First, basic demographic profiles, including age, sex, height, weight, and body mass index (BMI), were recorded. Additionally, various scoring systems for evaluating the medical and functional status of geriatric patients were employed. Activities of daily living (ADL), instrumental activities of daily living (IADL), and economic dependency were assessed, with nonzero values indicating functional dependency [14]. Short-form geriatric depression scale (GDS, range: 0–15) and mini-mental state examination (MMSE, range: 0–30) were used to assess the patient’s psychological state, with a GDS score of 5 or greater indicating depression and MMSE score of 23 or below suggesting cognitive impairment [15, 33]. Mini nutritional assessment (MNA, range: 0–30) was performed to assess the patient’s general nutritional status, with a higher score indicating more adequate nutrition [16]. As comorbidity status is crucial in evaluating elderly patients, CCI and the number of medications were also recorded [13]. Frailty measures, including the modified frailty index (mFI), timed-up-and-go test (TUGT) time in seconds, fall history within 6 months before surgery, and fracture due to fall within 6 months before surgery, were assessed [9].

Preoperative features such as ASA classification and laboratory test results were collected one day prior to surgery [12]. The laboratory tests included leukocyte count, hemoglobin, blood glucose, blood urea nitrogen (BUN), serum creatine, estimated glomerular filtration rate (eGFR), total cholesterol, serum protein, serum albumin, aspartate aminotransferase (AST), and alanine aminotransferase (ALT). Furthermore, features relevant to surgical plans, including spine fusion and the number of involved vertebra levels, were also considered as inputs. In total, 48 variables were collected from each patient. Comprehensive interviews and complete retrieval of test results were conducted for every individual, taking approximately one hour per patient, resulting in no missing data. Min-max scaling was applied to all of the values into a range between 0 and 1.

In this study, several outcome measures were predicted. First, postoperative complications occurring within 30 days were evaluated during the hospitalization period and at outpatient visits 1 month after surgery. These complications were categorized into general and surgical complications, with events classified as Clavien-Dindo grade 2 (complication requiring pharmacological treatment with drugs other than analgesics, antiemetics, antipyretics, diuretics, and electrolytes) or higher considered positive for postoperative complications [34]. Additionally, the need for transfusion during hospitalization was recorded as a binary variable, capturing any instance of red blood cell (RBC), fresh frozen plasma (FFP), or platelet transfusion. As a result, three outcome variables were studied in this research: (1) general or surgical postoperative complications within 30 days (2) general postoperative complications within 30 days (3) the need for transfusion within the hospitalization period.

### Model development

The patient cohort was randomly split into train-validation and test sets in a roughly 4:1 ratio, to ensure sufficient data for model training while preserving an independent set for final evaluation. Although the dataset was imbalanced, stratification and oversampling were not performed, as the same dataset was intended to be used for multiple tasks. Instead, to address the class imbalance during model training, a class weight ranging from 3 to 10 was assigned to the positive class, depending on the specific task and outcome distribution. To assess potential differences in input and output variables between the train-validation and test sets, statistical analyses were performed. Continuous variables were compared using independent t-tests, while categorical variables were analyzed using chi-square tests. P-values below 0.05 were considered statistically significant.

In this study, two types of predictive models were developed. The *Complex* model utilized all available input features collected, whereas the *Compact* model was designed to use a simplified set of 28 clinically relevant features, selected to eliminate redundancy in the input space and promote applicability for future studies. The predictive performance of both models was compared against the probability of “serious complication” as estimated by the ACS-NSQIP score, a widely recognized benchmark for predicting complications in non-cardiac surgery [11].

For each of the three outcome variables, five different machine learning algorithms were trained: logistic regression (LogReg), support vector machine (SVM) with radial basis function kernels, random forest classifier (RF), extreme gradient boosting (XGBoost) classifier, and light gradient boost machine (LightGBM) [20–22]. To maximize the utilization of available data, 5-fold cross-validation was applied to the train-validation set. Models were trained to minimize binary cross-entropy loss, and hyperparameter tuning was performed using Optuna (version 4.2.1), with 200 optimization trials conducted within a sufficiently broad hyperparameter search space, as detailed in Table 1 [35]. The combination of an extensive search space and a large number of trials was designed to ensure a thorough exploration of the parameter landscape and a high likelihood of converging to a near-optimal solution. The outputs from models trained in each fold were combined using soft voting to generate the final prediction probabilities.

**Table 1:** Hyperparameter search space

Hyperparameter	Search Space
**Logistic regression**	
Regularization constant	LogUniform(10^− 4^, 10^− 2^)
Regularization function	{ ElasticNet }
L1 to L2 ratio	Uniform(0, 1)
Class weight	Uniform(3, 10)
**Support vector machine**	
Regularization constant	LogUniform(10^− 4^, 10^− 2^)
Kernel	Radial basis function
Class weight	Uniform(3, 10)
**Random forest classifier**	
Number of estimators	Discrete(200, 1000, step = 100)
Splitting criterion	{ Gini index, Entropy }
Maximum tree depth	Discrete(5, 10, step = 1)
Minimum samples to split	Discrete(2, 10, step = 1)
Minimum samples per leaf	Discrete(1, 10, step = 1)
Proportion of features considerd for split	Discrete(0.2, 0.9, step = 1)
Class weight	Uniform(3, 10)
**XGBoost classifier**	
Tweedie variance power	Discrete(1.0, 2.0, step = 0.1)
Number of estimators	Discrete(200, 1000, step = 100)
Maximum tree depth	Discrete(5, 10, step = 1)
Learning rate	LogUniform(10^− 3^, 10^− 1^)
Proportion of features considerd for split	Discrete(0.2, 0.9, step = 0.1)
Subsampling ratio by trees	Discrete(0.2, 0.9, step = 0.1)
Subsampling ratio by level	Discrete(0.2, 0.9, step = 0.1)
Minimum child weight	LogUniform(10^− 4^, 10^4^)
L1 regularization coefficient	LogUniform(10^− 4^, 10^4^)
L2 regularization coefficient	LogUniform(10^− 4^, 10^4^)
Minimum loss reduction for split (gamma)	LogUniform(10^− 4^, 10^4^)
Early stopping rounds	Discrete(20, 40, step = 10)
Class weight	Uniform(3, 10)
**Light GBM**	
Tweedie variance factor	Discrete(1.0, 2.0, step = 0.1)
Number of estimators	Discrete(200, 1000, step = 100)
Maximum tree depth	Discrete(5, 10, step = 1)
Number of leaves	Discrete(31, 1000, step = 1)
Minimum samples per leaf	Discrete(1, 10, step = 1)
Learning rate	LogUniform(10^− 3^, 10^− 1^)
Proportion of features considerd for split	Discrete(0.2, 0.9, step = 0.1)
Bagging fraction	Discrete(0.2, 0.9, step = 0.1)
Bagging frequency	Discrete(1, 10, step = 1)
Subsampling ratio by trees	Discrete(0.2, 0.9, step = 0.1)
Subsampling ratio by level	Discrete(0.2, 0.9, step = 0.1)
L1 regularization coefficient	LogUniform(10^− 4^, 10^4^)
L2 regularization coefficient	LogUniform(10^− 4^, 10^4^)
Early stopping rounds	Discrete(20, 40, step = 10)
Class weight	Uniform(3, 10)

Hyperparameter search space was designed to ensure a thorough exploration of the parameter landscape and a high likelihood of converging to a near-optimal solution

### Evaluation of model performance

The area under the receiver-operating curve (AUROC) and the area under the precision-recall curve (AUPRC) on the test was used as the primary performance measure in this study, as these metrics are independent of the decision threshold. Additionally, several auxiliary metrics, including accuracy, sensitivity, specificity, positive predictive value (PPV), negative predictive value, and F1-score, were evaluated. They were calculated at Youden’s J point, a decision threshold that maximizes the sum of sensitivity and specificity [36].

First, the non-inferiority of the *Compact* model compared to the *Complex* model was statistically evaluated. Similarly, the performance of the *Compact* model was also compared against the ACS-NSQIP score, which served as a reference benchmark. In addition, performance differences among various machine learning algorithms within the *Compact* model framework were assessed. Since the dataset was relatively small, 95% confidence intervals for performance metrics, as well as their comparisons, were estimated using bootstrap resampling.

To assess the interpretability of the models, SHAP analysis was conducted on the *Compact* model. It evaluates the influence of each input feature on the final prediction, and a greater mean absolute SHAP value indicates a greater contribution to the model’s decision-making process [32]. For each trained model, the top 10 most influential features were identified, and their contributions were qualitatively examined using beeswarm plots generated from the test set.

## Results

### Cohort characteristics

A total of 278 patients enrolled in the study, and 17 were excluded due to the cancellation of surgery or change of treatment plans, resulting in 261 eligible for the study. The mean age of the cohort was 72.3 ± 4.8 years, and 108 (41.3%) patients were male. In total, 27 (10.3%) patients experienced either general or surgical postoperative complications of Clavien-Dindo grade 2 or higher within 30 days, with 20 (7.7%) general and 7 (2.7%) surgical. General complications consisted of 5 (1.9%) delirium, 5 (1.9%) cardiovascular, 4 (1.5%) urinary, 3 (1.1%) respiratory, 2 (0.8%) gastrointestinal, and 1 (0.4%) sacral fracture complications. Among surgical complications, neurologic symptoms were the most common, with 4 (1.5%) cases, followed by infection, hematoma, and pneumoperitoneum, each with 1 (0.4%) case. Transfusion was performed in 79 (30.2%) patients within the hospitalization period.

After the dataset was split, the train-validation and test sets contained 208 (79.7%) and 53 (20.3%) patients, respectively. Among the collected variables, only age (72.0 ± 4.8 vs. 73.5 ± 4.8 years; *P* = 0.049) and TUGT time (21.8 ± 44.0 vs. 13.7 ± 5.6 s; *P* = 0.02) showed significant differences. No evidence of a significant difference was found in general or surgical complication rates (10.6% vs. 9.4%; *P* = 1.00), general complication rates (7.7% vs. 7.5%; *P* = 1.00), and transfusion rates during hospitalization (31.7% vs. 24.5%; *P* = 0.39). A detailed comparison of all features between the train-validation and test sets is provided in Tables 2 and 3. The distribution of the output variable across the five cross-validation folds was examined using Fisher’s exact test, and no statistically significant differences were observed (*P* = 0.99, *P* = 0.72, and *P* = 0.98, respectively).

**Table 2:** Comparison of patient characteristics of the train-validation and test sets

Features	Train-validation set (*n* = 208)	Test set (*n* = 53)	*P*-value
**Demographic Information**			
Age	72.0 ± 4.8	73.5 ± 4.8	0.049*
Sex			0.49
Male	81 (39.7%)	24 (46.2%)	
Female	123 (60.3%)	28 (53.8%)	
Height (m)	1.6 ± 0.1	1.6 ± 0.1	0.44
Weight (kg)	61.8 ± 10.0	64.4 ± 10.4	0.11
BMI (kg/m^2^)	25.0 ± 3.0	25.6 ± 3.0	0.15
**Geriatric Assessment Tools**			
Activities of daily living (ADL)	7.6 ± 1.8	7.5 ± 1.7	0.67
Instrumental activities of daily living (IADL)	13.2 ± 4.3	12.9 ± 4.0	0.63
Geriatric depression scale (GDS) – short form	5.4 ± 3.7	6.5 ± 3.6	0.07
Mini-mental state exam (MMSE)	27.1 ± 2.3	27.0 ± 2.6	0.78
Charlson comorbidity index (CCI)	0.8 ± 1.2	1.2 ± 1.6	0.20
Mini-nutritional assessment (MNA)	22.2 ± 4.2	21.7 ± 4.3	0.53
Economic dependence			0.65
Independent	140 (67.3%)	38 (71.7%)	
Dependent	68 (32.7%)	15 (28.3%)	
Living dependence			0.75
Independent	146 (70.2%)	39 (73.6%)	
Dependent	62 (29.8%)	14 (26.4%)	
**Frailty Measures**			
Modified frailty index − 11 (mFI-11)	0.1 ± 0.1	0.1 ± 0.1	0.65
Modified frailty index − 5 (mFI-5)	1.2 ± 0.9	1.2 ± 0.8	0.63
Timed-up-and-go test (TUGT) (s)	21.8 ± 44.0	13.7 ± 5.6	0.02*
Fall history within 6 months			0.50
No	186 (89.4%)	45 (84.9%)	
Yes	22 (10.6%)	8 (15.1%)	
Fracture due to fall within 6 months			0.78
No	200 (96.2%)	52 (98.1%)	
Yes	8 (3.8%)	1 (1.9%)	

Continuous variables are shown in the format mean ± standard deviation, and independent t-test was performed to assess the difference in their distributions. Numbers in parentheses indicate the proportion of corresponding category, and the distribution of categorical variables are compared with chi-square test. P-values less than 0.05 indicate statistical significance and are marked with an asterisk (*). BMI, body mass index; ADL, activities of daily living; IADL, instrumental activities of daily living; GDS, geriatric depression scale; MMSE, mini-mental state exam; CCI, Charlson comorbidity index; MNA, mini-nutritional assessment; mFI, modified frailty index; TUGT, timed-up-and-go test

**Table 3:** Comparison of perioperative features between train-validation and test sets

Features	Train-validation set (*n* = 208)	Test set (*n* = 53)	*P*-value
**Preoperative Assessment**			
ASA classification			0.82
ASA 1	36 (17.3%)	9 (17.0%)	
ASA 2	155 (74.5%)	41 (77.4%)	
ASA 3	17 (8.2%)	3 (5.7%)	
Leukocyte count (x10^3^/mm^3^)	6.5 ± 1.7	6.9 ± 2.4	0.22
Hemoglobin (g/dL)	13.1 ± 1.6	13.1 ± 1.8	0.86
Blood glucose (mg/dL)	126.1 ± 40.9	127.9 ± 38.8	0.76
BUN (mg/dL)	17.5 ± 5.6	18.0 ± 5.5	0.57
Serum creatinine (mg/dL)	0.9 ± 0.5	0.9 ± 0.6	0.39
eGFR (mL/min)	83.0 ± 24.1	80.3 ± 19.9	0.41
Total cholesterol (mg/dL)	175.1 ± 42.5	171.2 ± 39.5	0.61
Serum protein (g/dL)	7.8 ± 7.1	8.2 ± 8.7	0.77
Serum albumin (g/dL)	4.3 ± 1.9	4.1 ± 0.3	0.30
AST (IU/L)	26.0 ± 16.9	25.2 ± 9.0	0.63
ALT (IU/L)	23.9 ± 18.9	23.4 ± 12.7	0.82
**Operation Characteristics**			
Spine fusion			0.08
No	44 (21.2%)	18 (34.0%)	
Yes	164 (78.8%)	35 (66.0%)	
Number of involved vertebra levels			0.40
1	100 (48.1%)	21 (39.6%)	
2	63 (30.3%)	21 (39.6%)	
3	45 (21.6%)	11 (20.8%)	
**Output Variables**			
General/surgical complications within 30 days			1.00
No	186 (89.4%)	48 (90.6%)	
Yes	22 (10.6%)	5 (9.4%)	
General complications within 30 days			1.00
No	192 (92.3%)	49 (92.5%)	
Yes	16 (7.7%)	4 (7.5%)	
Transfusion during hospitalization			0.39
No	142 (68.3%)	40 (75.5%)	
Yes	66 (31.7%)	13 (24.5%)	

Continuous variables are shown in the format mean ± standard deviation, and independent t-test was performed to assess the difference in their distributions. Numbers in parentheses indicate the proportion of corresponding category, and the distribution of categorical variables are compared with chi-square test. P-values less than 0.05 indicate statistical significance and are marked with an asterisk (*). ASA, American Society of Anesthesiologist Classification System; BUN, blood urea nitrogen; eGFR, estimated glomerular filtration rate; AST, aspartate aminotransferase; ALT, alanine aminotransferase

### Model performance

**Fig. 1:**
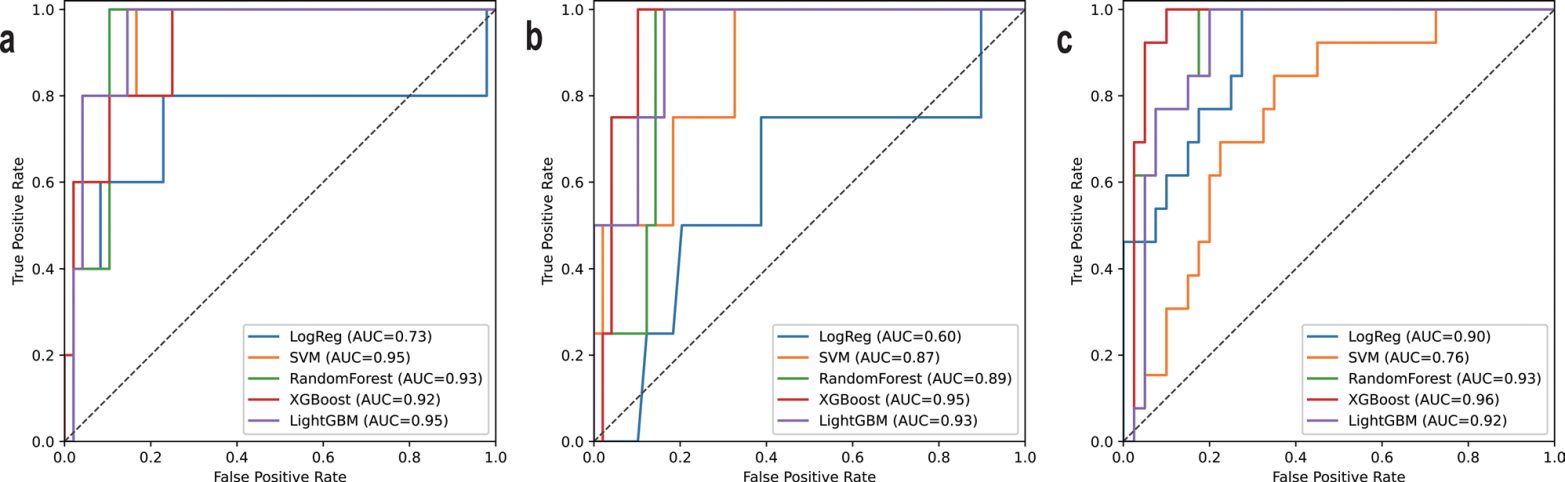
Receiver-operating characteristic curves on the test set (*Compact* model**).** Receiver-operating characteristic curves for predicting (**a**) early general and surgical complications, (**b**) early general complications, and (**c**) transfusion within hospitalization period. LogReg, logistic regression; SVM, support vector machine; AUC, area under the receiver-operating characteristic curve

The performance of the best models trained with each algorithm on each task is summarized in Tables 4, 5 and 6, and the receiver-operating characteristic (ROC) curve of the *Compact* model are plotted in Fig. 1.

**Table 4:** Performance on predicting general/surgical complications on the test set

Model	AUROC	AUPRC	Accuracy	Sensitivity	Specificity	PPV	NPV	F1-score
**Complex Model**								
LogReg	0.71 (0.30–0.96)	0.39 (0.07–0.78)	0.83 (0.72–0.92)	0.80 (0.33-1.00)	0.83 (0.72–0.94)	0.33 (0.08–0.63)	0.98 (0.92-1.00)	0.47 (0.14–0.73)
SVM	0.95 (0.86-1.00)	0.59 (0.26-1.00)	0.85 (0.75–0.94)	1.00 (1.00–1.00)	0.83 (0.72–0.93)	0.38 (0.13–0.67)	1.00 (1.00–1.00)	0.56 (0.22–0.80)
Random Forest	0.90 (0.81–0.98)	0.41 (0.16–0.85)	0.87 (0.77–0.94)	1.00 (1.00–1.00)	0.85 (0.75–0.94)	0.42 (0.15–0.71)	1.00 (1.00–1.00)	0.59 (0.27–0.83)
XGBoost	0.90 (0.77–0.98)	0.44 (0.14–0.88)	0.81 (0.70–0.91)	1.00 (1.00–1.00)	0.79 (0.67–0.90)	0.33 (0.11–0.60)	1.00 (1.00–1.00)	0.50 (0.19–0.75)
LightGBM	0.94 (0.86-1.00)	0.61 (0.21-1.00)	0.91 (0.83–0.98)	1.00 (1.00–1.00)	0.90 (0.80–0.98)	0.50 (0.20–0.83)	1.00 (1.00–1.00)	0.67 (0.33–0.91)
**Compact Model**								
LogReg	0.73 (0.32–0.99)	0.39 (0.09–0.91)	0.77 (0.66–0.89)	0.80 (0.33-1.00)	0.77 (0.65–0.89)	0.27 (0.07–0.53)	0.97 (0.91-1.00)	0.40 (0.12–0.67)
SVM	0.95 (0.87-1.00)	0.69 (0.29-1.00)	0.85 (0.75–0.94)	1.00 (1.00–1.00)	0.83 (0.73–0.93)	0.38 (0.13–0.67)	1.00 (1.00–1.00)	0.56 (0.24–0.80)
Random Forest	0.93 (0.84–0.99)	0.50 (0.20–0.95)	0.91 (0.81–0.98)	1.00 (1.00–1.00)	0.90 (0.80–0.98)	0.50 (0.20–0.83)	1.00 (1.00–1.00)	0.67 (0.33–0.91)
XGBoost	0.92 (0.80-1.00)	0.63 (0.21-1.00)	0.77 (0.66–0.87)	1.00 (1.00–1.00)	0.75 (0.62–0.86)	0.29 (0.10–0.53)	1.00 (1.00–1.00)	0.45 (0.18–0.69)
LightGBM	0.95 (0.87-1.00)	0.57 (0.25-1.00)	0.87 (0.77–0.96)	1.00 (1.00–1.00)	0.85 (0.74–0.95)	0.42 (0.15–0.73)	1.00 (1.00–1.00)	0.59 (0.27–0.84)
**ACS-NSQIP**	0.38 (0.13–0.73)	0.17 (0.03–0.58)	0.91 (0.83–0.96)	0.20 (0.00-0.67)	0.98 (0.93-1.00)	0.50 (0.00–1.00)	0.92 (0.84–0.98)	0.29 (0.00-0.73)

All performance metrics were measured on the test set, and all metrics except for AUROC and AUPRC were measured at Youden’s J point, a decision threshold that maximizes the sum of sensitivity and specificity. Numbers in parentheses indicate 95% confidence intervals. AUROC, area under the receiver-operating characteristic curve; AUPRC, area under the precision-recall curve; PPV, positive predictive value; NPV, negative predictive value

**Table 5:** Performance on predicting general complications on the test set

Model	AUROC	AUPRC	Accuracy	Sensitivity	Specificity	PPV	NPV	F1-score
**Complex Model**								
LogReg	0.63 (0.31–0.94)	0.18 (0.03–0.60)	0.40 (0.26–0.53)	1.00 (1.00–1.00)	0.35 (0.22–0.48)	0.11 (0.03–0.22)	1.00 (1.00–1.00)	0.20 (0.05–0.36)
SVM	0.67 (0.44-1.00)	0.34 (0.04-1.00)	0.53 (0.40–0.66)	1.00 (1.00–1.00)	0.49 (0.35–0.63)	0.14 (0.03–0.27)	1.00 (1.00–1.00)	0.24 (0.06–0.42)
Random Forest	0.88 (0.74–0.98)	0.32 (0.10–0.80)	0.77 (0.66–0.89)	1.00 (1.00–1.00)	0.76 (0.63–0.87)	0.25 (0.07–0.47)	1.00 (1.00–1.00)	0.40 (0.13–0.64)
XGBoost	0.94 (0.87-1.00)	0.47 (0.20-1.00)	0.91 (0.83–0.98)	1.00 (1.00–1.00)	0.90 (0.81–0.98)	0.44 (0.14–0.80)	1.00 (1.00–1.00)	0.62 (0.25–0.89)
LightGBM	0.92 (0.83–0.99)	0.42 (0.17–0.95)	0.87 (0.77–0.94)	1.00 (1.00–1.00)	0.86 (0.75–0.94)	0.36 (0.11–0.67)	1.00 (1.00–1.00)	0.53 (0.20–0.80)
**Compact Model**								
LogReg	0.60 (0.13–0.89)	0.13 (0.04–0.38)	0.62 (0.49–0.75)	0.75 (0.00–1.00)	0.61 (0.48–0.75)	0.14 (0.00-0.29)	0.97 (0.89-1.00)	0.23 (0.00-0.44)
SVM	0.87 (0.68-1.00)	0.53 (0.08-1.00)	0.70 (0.58–0.81)	1.00 (1.00–1.00)	0.67 (0.54–0.80)	0.20 (0.05–0.39)	1.00 (1.00–1.00)	0.33 (0.10–0.56)
Random Forest	0.89 (0.79–0.98)	0.35 (0.11–0.82)	0.87 (0.77–0.96)	1.00 (1.00–1.00)	0.86 (0.76–0.95)	0.36 (0.11–0.67)	1.00 (1.00–1.00)	0.53 (0.20–0.80)
XGBoost	0.95 (0.88-1.00)	0.51 (0.20-1.00)	0.91 (0.83–0.98)	1.00 (1.00–1.00)	0.90 (0.81–0.98)	0.44 (0.14–0.78)	1.00 (1.00–1.00)	0.62 (0.25–0.88)
LightGBM	0.93 (0.82-1.00)	0.68 (0.14-1.00)	0.85 (0.74–0.94)	1.00 (1.00–1.00)	0.84 (0.72–0.93)	0.33 (0.09–0.63)	1.00 (1.00–1.00)	0.50 (0.17–0.77)
**ACS-NSQIP**	0.22 (0.10–0.36)	0.06 (0.02–0.13)	0.17 (0.08–0.28)	1.00 (1.00–1.00)	0.10 (0.02–0.19)	0.08 (0.02–0.17)	1.00 (1.00–1.00)	0.15 (0.04–0.29)

All performance metrics were measured on the test set, and all metrics except for AUROC and AUPRC were measured at Youden’s J point, a decision threshold that maximizes the sum of sensitivity and specificity. Numbers in parentheses indicate 95% confidence intervals. AUROC, area under the receiver-operating characteristic curve; AUPRC, area under the precision-recall curve; PPV, positive predictive value; NPV, negative predictive value

**Table 6:** Performance on predicting transfusion within hospitalization period on the test set

Model	AUROC	AUPRC	Accuracy	Sensitivity	Specificity	PPV	NPV	F1-score
**Complex Model**								
LogReg	0.90 (0.81–0.97)	0.79 (0.58–0.94)	0.81 (0.70–0.91)	1.00 (1.00–1.00)	0.75 (0.61–0.88)	0.57 (0.35–0.77)	1.00 (1.00–1.00)	0.72 (0.52–0.87)
SVM	0.77 (0.62–0.89)	0.46 (0.28–0.74)	0.72 (0.60–0.83)	0.92 (0.73-1.00)	0.65 (0.50–0.78)	0.46 (0.28–0.65)	0.96 (0.88-1.00)	0.62 (0.42–0.77)
Random Forest	0.93 (0.85–0.98)	0.78 (0.55–0.96)	0.85 (0.75–0.94)	0.92 (0.75-1.00)	0.83 (0.70–0.93)	0.63 (0.41–0.84)	0.97 (0.91-1.00)	0.75 (0.56–0.90)
XGBoost	0.92 (0.84–0.98)	0.81 (0.60–0.95)	0.87 (0.77–0.94)	0.92 (0.75-1.00)	0.85 (0.73–0.95)	0.67 (0.44–0.88)	0.97 (0.91-1.00)	0.77 (0.58–0.92)
LightGBM	0.92 (0.83–0.98)	0.74 (0.50–0.96)	0.91 (0.83–0.98)	0.85 (0.63-1.00)	0.93 (0.83-1.00)	0.79 (0.55-1.00)	0.95 (0.87-1.00)	0.81 (0.63–0.95)
**Compact Model**								
LogReg	0.90 (0.80–0.97)	0.78 (0.56–0.93)	0.79 (0.68–0.91)	1.00 (1.00–1.00)	0.73 (0.58–0.86)	0.54 (0.33–0.75)	1.00 (1.00–1.00)	0.70 (0.50–0.86)
SVM	0.76 (0.61–0.89)	0.45 (0.27–0.74)	0.70 (0.58–0.81)	0.85 (0.62-1.00)	0.65 (0.50–0.79)	0.44 (0.26–0.64)	0.93 (0.81-1.00)	0.58 (0.38–0.74)
Random Forest	0.93 (0.86–0.99)	0.74 (0.52–0.97)	0.87 (0.77–0.94)	1.00 (1.00–1.00)	0.83 (0.70–0.93)	0.65 (0.43–0.86)	1.00 (1.00–1.00)	0.79 (0.60–0.92)
XGBoost	0.96 (0.90-1.00)	0.80 (0.58-1.00)	0.92 (0.85–0.98)	1.00 (1.00–1.00)	0.90 (0.79–0.98)	0.76 (0.55–0.94)	1.00 (1.00–1.00)	0.87 (0.71–0.97)
LightGBM	0.92 (0.83–0.98)	0.67 (0.45–0.96)	0.85 (0.75–0.94)	1.00 (1.00–1.00)	0.80 (0.68–0.92)	0.62 (0.41–0.83)	1.00 (1.00–1.00)	0.76 (0.58–0.91)

All performance metrics were measured on the test set, and all metrics except for AUROC and AUPRC were measured at Youden’s J point, a decision threshold that maximizes the sum of sensitivity and specificity. Numbers in parentheses indicate 95% confidence intervals. AUROC, area under the receiver-operating characteristic curve; AUPRC, area under the precision-recall curve; PPV, positive predictive value; NPV, negative predictive value

Compared to the *Complex* model, the *Compact* model did not demonstrate statistically significant inferiority in terms of AUROC or AUPRC for any of the tested machine learning algorithms. Furthermore, when compared to the ACS-NSQIP scoring system, which achieved a test AUROC of 0.38 (95% confidence interval [CI], 0.13–0.73) and 0.22 (95% CI, 0.10–0.36) on the first two tasks, the *Compact* model showed significantly greater AUROC values nearing or surpassing 0.90 for all algorithms except for LogReg (*P* = 0.10 and *P* = 0.61, respectively for each task). Although the *Compact* model generally yielded higher AUPRC values as well, statistical significance was observed only in the case of the SVM trained on the first task (*P* = 0.044).

Overall, decision tree-based algorithms, including RF, XGBoost, and LightGBM, consistently demonstrated greater performance than LogReg, while the performance of SVMs fluctuated depending on the task. For LogReg, only XGBoost for the second task—predicting general postoperative complications within 30 days—showed statistically significantly higher performance, with AUROC (0.60 vs. 0.95; *P* = 0.03) and AUPRC (0.13 vs. 0.51; *P* = 0.04). In the other tasks, no statistically significant improvements over LogReg were observed. The superiority of tree-based models over SVM was shown in the task of predicting transfusion. In terms of AUROC, all tree-based models demonstrated statistically significantly higher performance: RF (0.76 vs. 0.93; *P* = 0.03), XGBoost (0.76 vs. 0.96; *P* = 0.005), and LightGBM (0.76 vs. 0.92; *P* = 0.049). For AUPRC, a statistically significant improvement was observed only with XGBoost (0.45 vs. 0.80; *P* = 0.04).

### Explainability analysis

Table 7 lists the top 10 most influential features of the *Compact* model for each ML algorithm identified by SHAP analysis. The results indicate that the key contributing factors were generally consistent across different algorithms, particularly among decision tree-based models. For early postoperative complications, important predictors included frailty measures such as fall history within 6 months and TUGT time, as well as preoperative laboratory test results and fusion operation. In the case of transfusion prediction, hemoglobin level, nutrition, and involved spinal levels were identified as major contributing factors.

**Table 7:** List of top 10 contributing features of the *Compact* model for each algorithm and task

LogReg	SVM	RF	XGBoost	LightGBM
**General or surgical complications within 30 days**				
TUGT time Total cholesterol MNA (full) BUN Leukocyte count Height Age BMI Serum creatinine Weight	Fall history (6 mo.) Height Total cholesterol TUGT time Leukocyte count Uncomplicated DM Hemoglobin Fusion operation Dependent economy eGFR	Glucose Height AST MNA (full) Fall history (6 mo.) Hemoglobin Total cholesterol Leukocyte count TUGT time Fusion operation	Fusion operation Height Fall history (6 mo.) Serum creatinine Total cholesterol Hemoglobin Dependent living Glucose Leukocyte count AST	AST TUGT time Hemoglobin MNA(full) BMI Fusion operation Height BUN Glucose Leukocyte count
**General complications within 30 days**				
Total cholesterol Glucose TUGT time Hemoglobin Serum albumin Spine level count Height Age BMI MNA (screen)	Height Total cholesterol Fall history (6 mo.) Serum creatinine TUGT time BMI eGFR Uncomplicated DM BUN ALT	Fusion operation Total cholesterol Dependent economy Dependent living AST MNA (full) Uncomplicated DM Hemoglobin Serum creatinine eGFR	Fusion operation Dependent living Total cholesterol Uncomplicated DM Height Complicated DM Glucose Fall history (6 mo.) Serum creatinine Sex	TUGT time Dependent living Fall history (6 mo.) MNA (full) BUN Dependent economy Glucose Height Hemoglobin BMI
**Transfusion during hospitalization**				
Height Total cholesterol Hemoglobin TUGT time Leukocyte count Fusion operation Weight Sex Hypertension BUN	Height TUGT time Fall history (6 mo.) Total cholesterol Hemogrlbin Leukocyte count Fusion operation Hypertension Uncomplicated DM Fall fracture history	Hemoglobin Spine level count MNA (full) Sex Leukocyte count TUGT time Height Weight eGFR Fusion operation	Hemoglobin Spine level count MNA (full) TUGT time Leukocyte count Uncomplicated DM Hypertension Weight Total cholesterol Age	Hemoglobin Spine level count MNA (full) TUGT time Height Weight Total cholesterol eGFR Sex MNA (screen)

The listed features are sorted in descending order according to the mean absolute Shapley values on the test set. ADL, activities of daily living; ALT, alanine aminotransferase; AST, aspartate aminotransferase; BUN, blood urea nitrogen; DM, diabetes mellitus; eGFR, estimated glomerular filtration rate; GDS, geriatric depression scale; LightGBM, light gradient boosing machine; LogReg, logistic regression; MNA, mini-nutritional assessment; SVM, support vector machine; RF, random forest classifier; TUGT, timed-up-and-go test

**Fig. 2:**
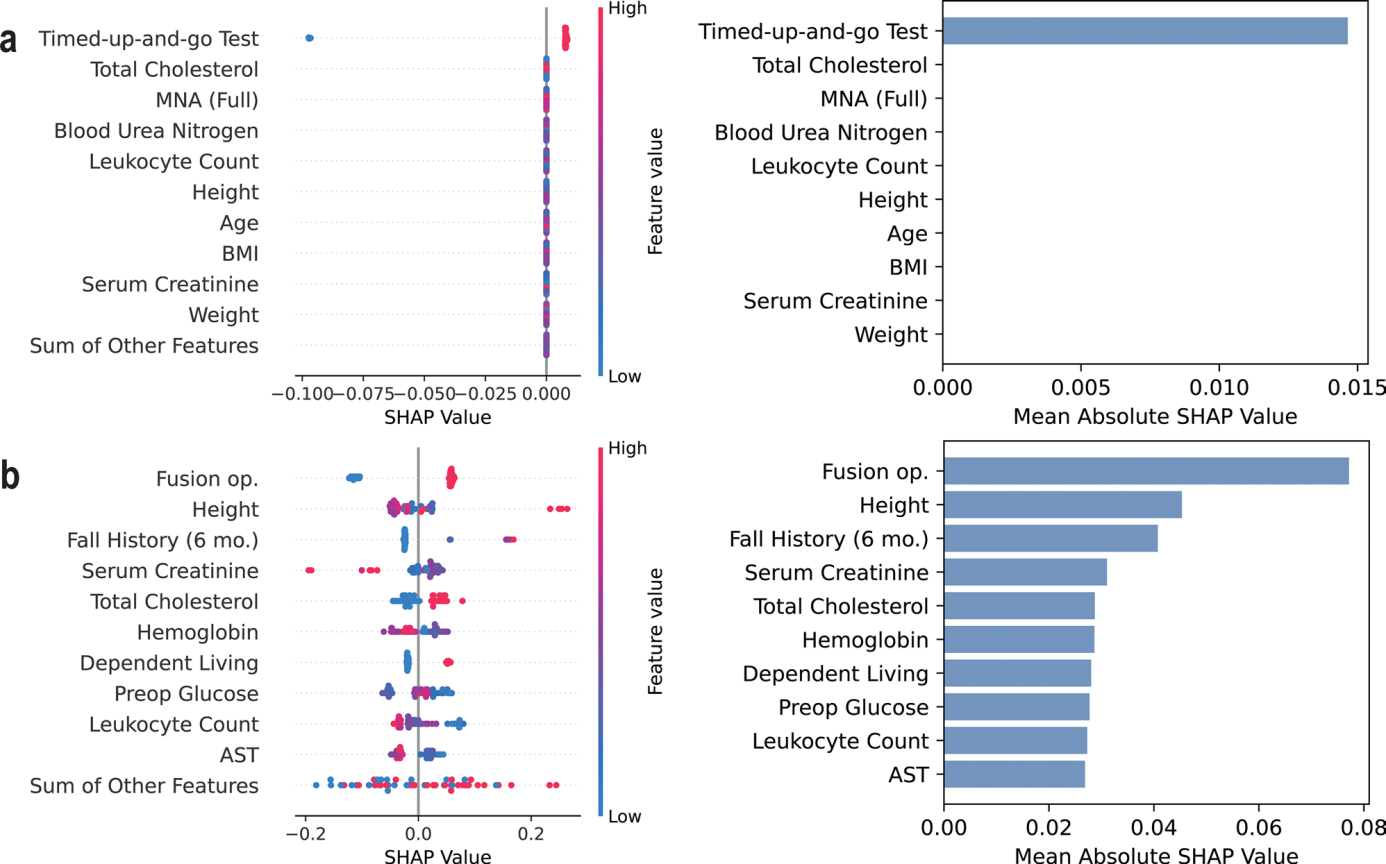
SHAP results on predicting early general and surgical postoperative complications (*Compact* model). Shapley additive explanation analysis results for (**a**) the logistic regression model and (**b**) XGBoost classifier on predicting early general and surgical postoperative complications

**Fig. 3:**
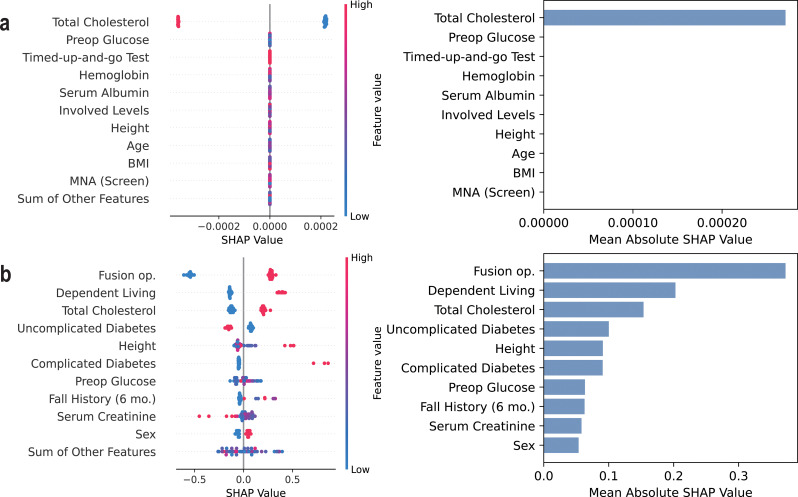
SHAP results on predicting early general postoperative complications (*Compact* model). Shapley additive explanation analysis results for (**a**) the logistic regression model and (**b**) XGBoost classifier on predicting early general postoperative complications

**Fig. 4:**
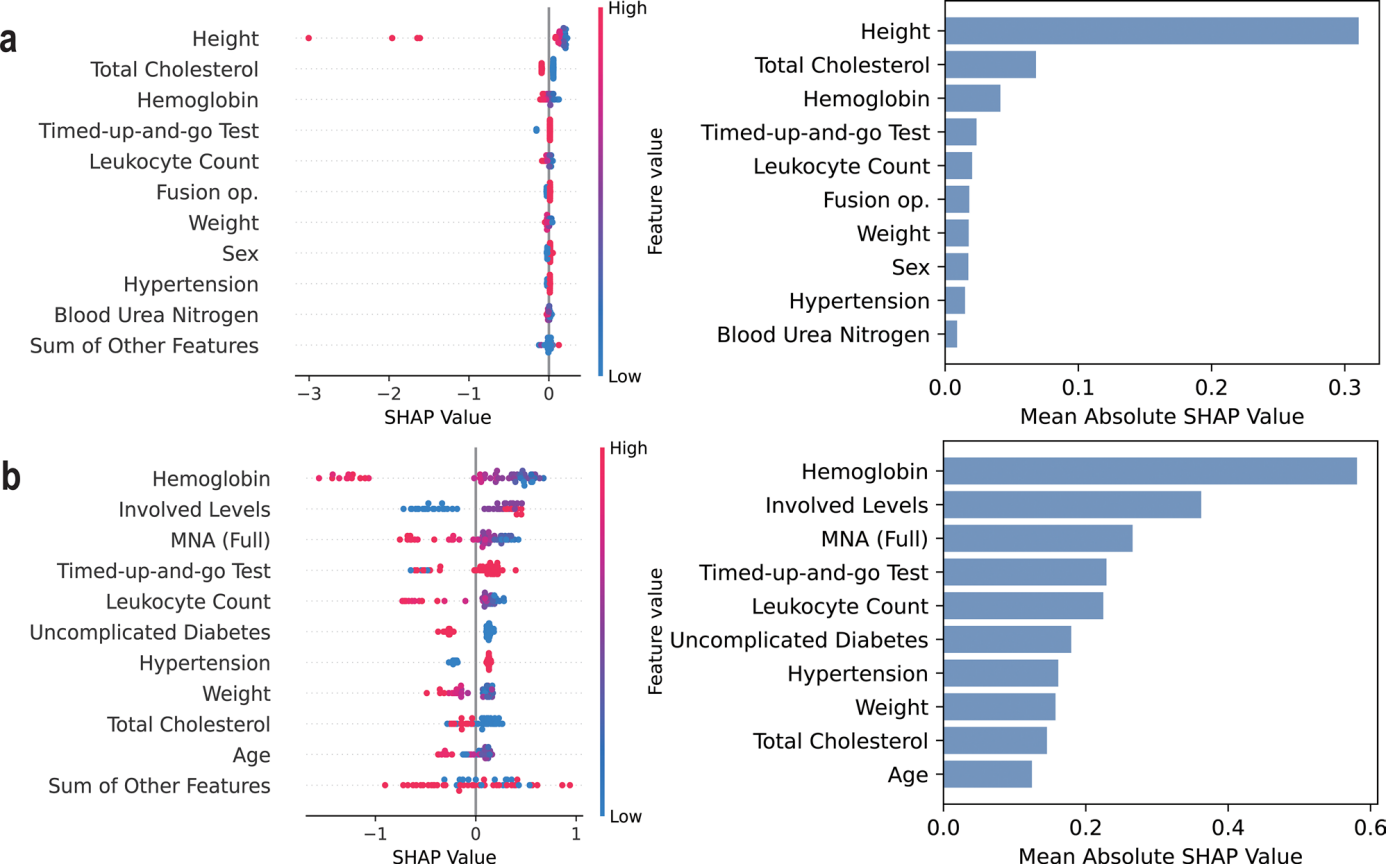
SHAP results on predicting transfusion during hospital stay (*Compact* model). Shapley additive explanation analysis results for (**a**) the logistic regression model and (**b**) XGBoost classifier on predicting transfusion within hospital stay

Figures 2, 3 and 4 present the beeswarm plots and mean absolute SHAP values obtained from each task, comparing LogReg and XGBoost. As demonstrated in Figs. 2a, 3a, and 4a, LogReg consistently exhibited poor group separation in the beeswarm plots, with SHAP values concentrated in only a few variables. Contrastincly, XGBoost demonstrated clear group separation across all tasks, with SHAP values more evenly distributed across multiple variables, as illustrated in Figs. 2b, 3b, and 4b. This suggests that XGBoost exhibits greater interpretability than LogReg. SHAP analysis results for SVM, random forest, and LightGBM are provided in the additional figures dedicated for demonstrating SHAP results, and decision tree-based models like random forest and LightGBM have shown similar patterns to XGBoost (see Additional files 1, 2, and 3).

## Discussion

In clinical research, statistical analysis methods have gradually shifted from traditional approaches, such as LogReg, to ML techniques due to their capability to capture nonlinear complex relationships in high-dimensional data [17, 18]. This shift is particularly relevant in the management of elderly patients, where multiple interrelated factors must be considered to achieve optimal outcomes [2, 5, 37, 38]. Accordingly, we applied ML to analyze our prospectively collected cohort, aiming to predict early postoperative complications and transfusion requirements in elderly patients undergoing surgery for LSS.

### Comparison between models

As demonstrated earlier, the ML models consistently outperformed the reference method, ACS-NSQIP, in both AUROC and AUPRC. In particular, for the first two tasks involving the prediction of early postoperative complications, ACS-NSQIP yielded AUROC values below 0.5, indicating substantially limited predictive capability. It should be noted that AUROC and AUPRC were adopted as the primary evaluation metrics because they are independent of the decision threshold. Although auxiliary metrics, such as accuracy, sensitivity, and specificity, were measured at Youden-J point, careful selection of the decision threshold is warranted, with due consideration of the potential consequences associated with false predictions.

No evidence of statistically significant differences in AUROC and AUPRC was observed between the *Complex* and the *Compact* model. In other words, the exclusion of time- and labor-intensive variables such as ADL, IADL, MMSE, and CCI did not compromise predictive performance. These findings suggest that the *Compact* model may offer a more practical and scalable approach for future research and clinical implementation. Nevertheless, much validation and clinical studies must be preceded before real application.

When comparing the performance across ML algorithms, decision tree–based models such as XGBoost and LightGBM generally outperformed others, particularly in the task of predicting transfusion, where they showed superior performance compared to LogReg and SVM. Of particular note are the SHAP analysis results, which illustrate model interpretability: while LogReg tended to rely heavily on a single variable, tree-based models distributed importance more evenly across multiple variables, reflecting a more holistic consideration of diverse clinical features. Considering both predictive performance and interpretability, models such as XGBoost and LightGBM appear to be more suitable for clinical application.

### Clinical implications

This section discusses the clinical implications and potentially actionable insights derived from the SHAP analysis of the *Compact* model based on RF, XGBoost, and LightGBM algorithms. By examining the contribution of individual features to the model’s predictions, we aim to identify clinically relevant patterns and risk factors that may inform preoperative decision-making and patient-specific care strategies.

First, frailty-related indicators, such as fall history within six months, and TUGT time, were one of the major contributing factors for early postoperative complications. This finding aligns with previous studies and highlights the importance of assessing frailty and muscle waste in elderly patients before lumbar spine surgery [9, 10, 39, 40]. Dependency in daily living and poor nutritional status were also associated with a greater risk of early postoperative complications and transfusion, highlighting the need for perioperative fitness for better recovery [5, 41]. Therefore, in patients with multiple risk factors, it would be prudent to actively consider non-surgical treatment options first and reserve surgical intervention only for cases with absolute indications, such as the occurrence of neurological complications.

Interestingly, age and comorbidity burden were found to be less influential than expected, with only diabetes showing notable contributions to the outcome variables [42, 43]. Instead, preoperative cholesterol, kidney-related markers, and nutritional status played more significant roles, suggesting the importance of effective management of comorbidities in the geriatric population [41, 44]. Specifically, cholesterol levels may have emerged as a contributing factor probably due to their link with cardiovascular health [45]. This finding emphasizes the importance of lipid management, even in patients without a history of cardiovascular disease, as a potentially actionable strategy to mitigate surgical risks in elderly patients [46–48].

Regarding transfusion risk, preoperative hemoglobin level was identified as a primary determinant, which is consistent with contemporary clinical practices and literature [29, 49]. Furthermore, patients who underwent procedures involving multiple vertebral levels, had a higher likelihood of requiring transfusion during hospitalization. Poor nutritional status and prolonged TUGT time was also associated with increased risk, underscoring the importance of preoperative nutritional management [34, 50, 51]. Thus, sufficient correction of nutrition should be considered prior to elective LSS in elderly patients to reduce the likelihood of transfusion events.

### Limitations and future work

This section outlines the limitations of the study and directions for future work. First, although statistical analyses have demonstrated that the developed ML models outperformed the ACS-NSQIP score in terms of AUROC, the generalizability of the findings is limited by the relatively small sample size (*n* = 261). The single-center design without external validation and the class imbalance in the dataset may have introduced considerable bias. Furthermore, the study included only patients who underwent elective surgery, raising concerns about potential selection bias. It is also possible that clinical decisions made by healthcare providers based on ASA classification or surgical planning may have led to an underestimation of the actual risk, further limiting the objectivity of the outcome labels.

The limited dataset size was largely due to the prospective enrollment of elderly patients and the extensive time and resources required to collect a wide range of questionnaire-based features. In particular, constructing the input for the *Complex* model necessitated a minimum of one hour of thorough, face-to-face interviews per patient to obtain data such as ADL, IADL, MNA, MMSE, and CCI. To address the associated burden, we developed the *Compact* model using only routinely available variables such as blood test results and clinically essential features. Notably, statistical analyses revealed no significant inferiority in predictive performance compared to the *Complex* model.

Therefore, this study not only demonstrates the value of ML in the proposed tasks as a proof-of-concept but also lays the groundwork for scalable and cost-effective research in larger cohorts. Although immediate clinical application may be limited, we believe that, with the development of multi-institutional, multiethnic datasets and sufficient validation through clinical trials, this approach holds promise for enabling individualized care for the geriatric population. Further integration into electronic health records or regulatory approval should also be followed.

Another downside of this study lies in the restricted scope of output variables. Due to the relatively small dataset, it was not feasible to develop models capable of predicting organ-specific complications. With a larger cohort in future research, such granularity may become attainable, allowing the model not only to assess the overall risk of complications but also to identify specific systems or organs that require heightened clinical attention. Additionally, in the geriatric population, long-term outcomes such as morbidity and mortality are of particular clinical relevance. The inability to evaluate these outcomes within the current study further limits its scope. Future investigations should therefore aim to expand not only the sample size but also the follow-up period to comprehensively assess both short- and long-term risks.

## Conclusion

ML offers a data-driven approach that can integrate numerous factors and capture intricate, nonlinear relationships among them. In this study, we have developed ML algorithms that predict early postoperative complications and transfusion within the hospitalization period in elderly patients undergoing elective surgery for LSS and observed that they can provide higher predictive performance than well-established scoring systems such as ACS-NSQIP. Although the scope of the study is limited by the small cohort collected from a single institution, it may serve as a foundation for future larger-scale reasearch and hopefully clinical application.

## Electronic supplementary material

Below is the link to the electronic supplementary material.

Supplementary Material 1

Supplementary Material 2

Supplementary Material 3

## Data Availability

The dataset generated and analyzed during the current study is not publicly available due to patient privacy concerns and ethical restrictions but are available from the corresponding author upon reasonable request.

## Abbreviations

ALT: Alanine aminotransferase
ASA: American Society of Anesthesiologist Physical Classification System
AST: Aspartate aminotransferase
AUPRC: Area under the precision-recall curve
AUROC: Area under the receiver-operating characteristic curve
ADL: Activities of daily living
BMI: Body mass index
BUN: Blood urea nitrogen
CCI: Charlson comorbidity index
eGFR: Estimated glomerular filtration rate
GDS: Geriatric depression scale
IADL: Instrumental activities of daily living
LightGBM: Light gradient boosted machine
LSS: Lumbar spinal stenosis
mFI: Modified frailty index
ML: Machine learning
MMSE: Mini-mental state examination
MNA: Mini nutritional assessment
NPV: Negative predictive value
PPV: Positive predictive value
RF: Random forest classifier
ROC: Receiver-operating characteristic
SHAP: Shapley additive explanations
SVM: Support vector machine
TUGT: Timed-up-and-go test
XGBoost: Extreme gradient boosting
