# Supplementary material for: Prediction of early postoperative complications and transfusion risk after lumbar spinal stenosis surgery in geriatric patients: machine learning approach based on comprehensive geriatric assessment
Source: BMC Med Inform Decis Mak. 2025 Jul 28;25:279. doi: 10.1186/s12911-025-03125-1 (PMC12306017; doi:10.1186/s12911-025-03125-1)
Supplement: Supplementary file 1 — Supplementary Material 1 [file 12911_2025_3125_MOESM1_ESM.docx]

**File name:** AdditionalFile_1.docx

**File format:** MS Word

**Title of data:** SHAP analysis results on predicting general or surgical postoperative complications within 30 days using *Compact* model.


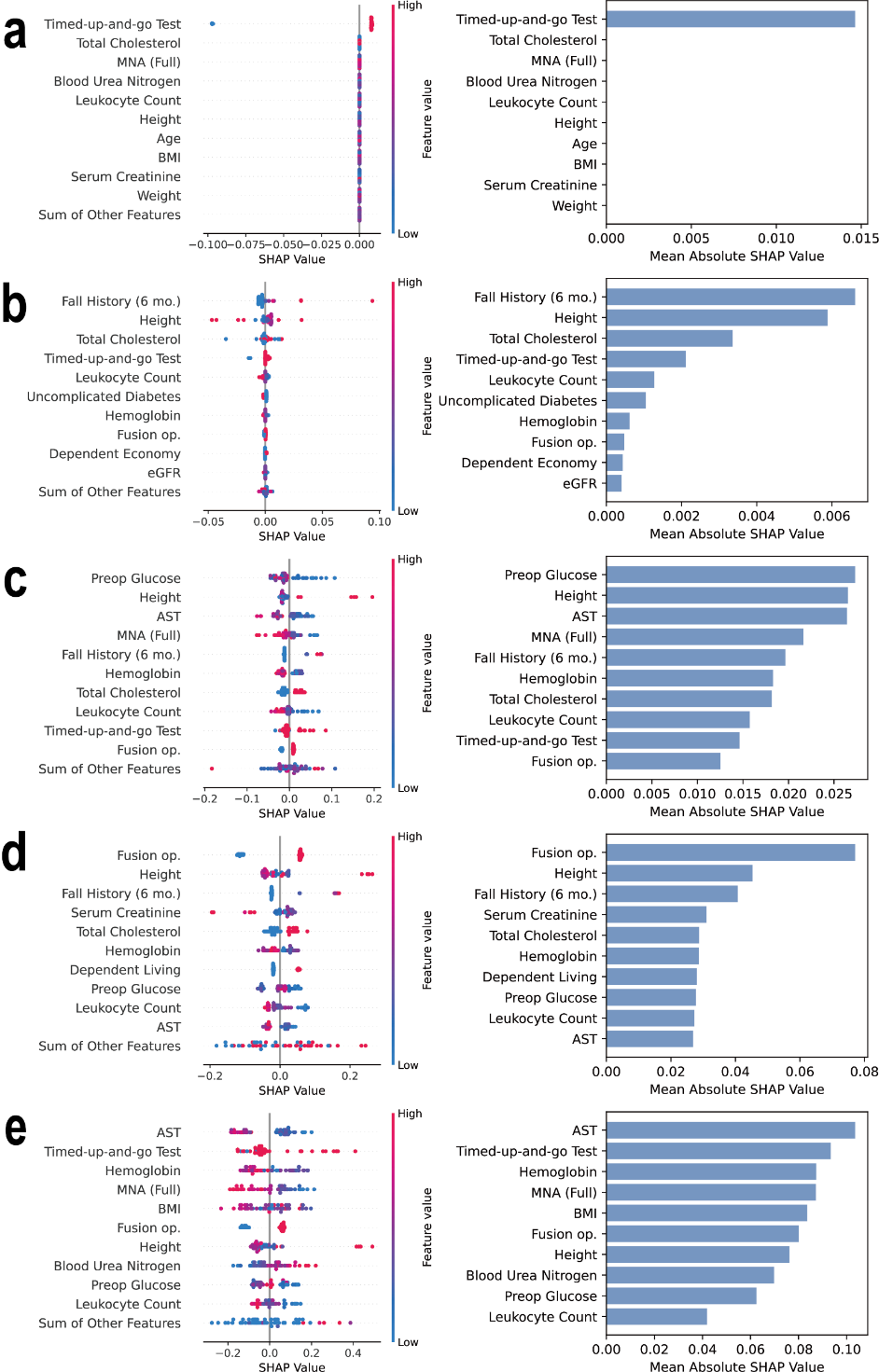


**Description of data:** Results of (a) logistic regression, (b) support vector machine, (c) random forest classifier, (d) XGBoost, and (e) LightGBM are demonstrated.
